# Cardiac autonomic function in patients with single ventricle physiology after Fontan palliation: A literature review

**DOI:** 10.1016/j.ijcchd.2025.100636

**Published:** 2025-10-30

**Authors:** Elizaveta Polyakova, Marieke Nederend, Philippine Kies, Anastasia D. Egorova, Monique R.M. Jongbloed

**Affiliations:** a CAHAL, Center for Congenital Heart Disease Amsterdam Leiden, Leiden University Medical Center, Leiden, the Netherlands; b Department of Anatomy & Embryology, Leiden University Medical Center, Leiden, the Netherlands; c Department of Cardiology, Leiden University Medical Center, Leiden, the Netherlands

**Keywords:** Congenital heart disease, Fontan circulation, Univentricular heart, Single ventricle disease, Heart rate variability, Cardiac autonomic nervous system

## Abstract

**Background:**

The Fontan operation has significantly improved survival in patients with single ventricle physiology. This comes at a price of highly prevalent long-term complications. Autonomic dysfunction has been documented in patients after Fontan palliation. Although autonomic dysfunction is associated with a range of adverse outcomes, the exact clinical implications in patients with a Fontan circulation remain unclear.

**Aims:**

The aims of this review are to address (1) the extent and characteristics of autonomic dysfunction in Fontan patients; (2) its association with clinical short- and long-term outcomes, and (3) identify key gaps in the literature.

**Methods:**

A literature search was performed in PubMed using a dedicated query for single ventricle disease and cardiac autonomic (dys)function.

**Results:**

Data from the literature consistently indicated overt autonomic dysfunction in patients after Fontan operation versus controls, marked by reduced heart rate variability and impaired parasympathetic tone. However, autonomic dysfunction showed different associations with clinical outcomes in Fontan patients. Several studies reported an association with diminished exercise capacity, arrhythmias, organ function markers, while other studies found no clear predictive value. Gaps in literature include data on adult patients, the influence of anatomical substrate, sex and lifestyle, as well as specific “driving” factors for the development of cardiac autonomic dysfunction.

**Conclusion:**

Autonomic dysfunction in Fontan patients is a persistent and potentially progressive condition, characterized by reduced heart rate variability and reduced parasympathetic tone. Long-term studies are needed to clarify its clinical significance and address gaps in literature relating to contributing factors like sex, anatomy, and underlying mechanisms.

## Highlights

• Fontan patients show persistent autonomic dysfunction with reduced HRV
• Autonomic dysfunction progresses through Glenn and TCPC surgical stages
• Impaired CANA is associated with lower exercise capacity and arrhythmia risk
• The effect of anatomy, sex, and lifestyle factors on CANA impairment remains limited
• Larger longitudinal studies are needed to clarify outcomes and prognostic value

## Graphical abstract

**Figure:**
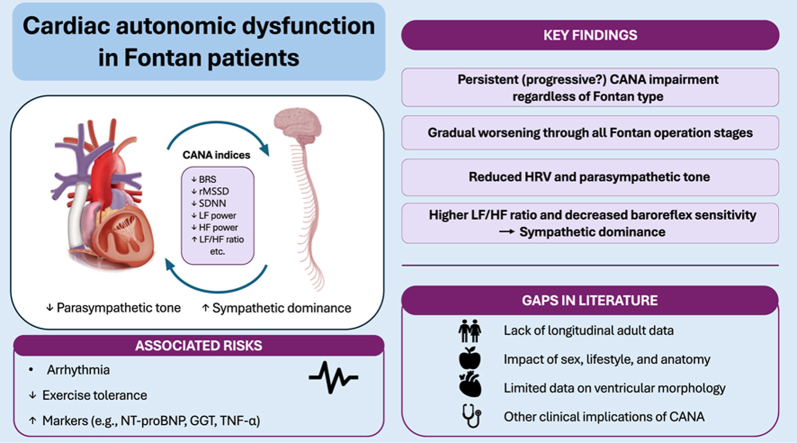

## 1 Introduction

**Fig. 1:**
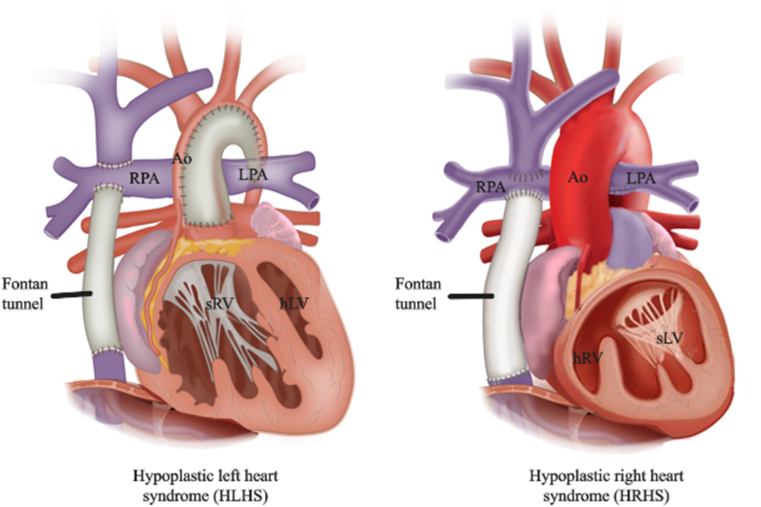
Schematic representation of Fontan tunnel in univentricular circulation in hypoplastic left heart syndrome (HLHS) and hypoplastic right heart syndrome (HRHS). *Left panel is modified from Smit et al.* [47]. *Right panel is modified from van der Woude* et al. [46] Ao: Aorta, RPA: right pulmonary artery, LPA: left pulmonary artery, hLV: hypoplastic left ventricle, hRV: hypoplastic right ventricle, sLV: systemic left ventricle, sRV: systemic right ventricle.

Congenital heart disease (CHD) is the most common congenital defect, affecting almost 1 in 100 new-borns [1]. As a result of improvements in surgery and healthcare, most patients are now living into adulthood, leading to a rapidly growing community of adults with congenital heart disease (ACHD). One of the most challenging groups of CHD patients is those with univentricular physiology after Fontan palliation (further referred to as “Fontan patients”) (Fig. 1). This morphologically heterogenous group consists of patients in whom the heart is not equipped to support a biventricular circulation. The Fontan procedure is a palliative surgical intervention that establishes a direct, passive pathway for the systemic venous return to the pulmonary circulation, thereby eliminating the reliance on a subpulmonary ventricle [[2], [3], [4]]. This fragile circulation is sustained by chronically elevated systemic venous pressures. Since its introduction in 1971, the Fontan procedure has significantly improved survival rates in patients with single ventricle physiology [2]. As a result, an increasing number of patients are now surviving into adulthood, leading to a growing population of adult Fontan survivors. However, this improved survival is associated with a high incidence of long-term, multisystem complications. By 40 years of age, only 41 % of patients remain free from major adverse events [[5], [6], [7], [8], [9], [10], [11], [12]]. Premature mortality remains prevalent in this population, although predicting individual clinical outcomes remains a significant challenge [6].

A common denominator of some of the late complications observed in patients with single ventricle physiology, might be autonomic dysfunction, which has been reported by a myriad of studies [[13], [14], [15]]. The autonomic nervous system, consisting of sympathetic and parasympathetic branches, is essential for cardiac innervation and the preservation of cardiovascular homeostasis throughout life. It facilitates physiological adaptation to different levels of activity through regulating heart rhythm and function. Parasympathetic activity is typically associated with cardioprotective influence, while sympathetic activation causes a cardio-stimulatory effect [16]. A shift towards elevated sympathetic tone is a well-recognized phenomena in the context of structural heart disease. This imbalance has been associated with adverse clinical outcomes, such as ventricular and atrial arrhythmias, progression of heart failure, and increased mortality [[17], [18], [19]].

Cardiac autonomic nervous activity (CANA) can be assessed by non-invasive testing, for example, Holter monitoring and heart rate variability (HRV) spectral analysis. Disturbed parameters of CANA have been associated with complications, such as arrhythmias in complex CHD patients [20,21]. Strikingly, especially in adult Fontan patients, who have a high incidence of arrhythmias and heart failure, the data on the relationship between (impaired) CANA and clinical outcomes is scarce and inconclusive [14,[22], [23], [24], [25]].

Hence, this literature review aims to explore existing evidence on cardiac autonomic dysfunction and its association with outcomes in pediatric and adult Fontan patients, while highlighting important gaps in understanding its underlying determinants.

## 2 Methods

### 2.1 Literature review

#### 2.1.1 Search strategy

A comprehensive narrative literature review was performed in June 2025 using the search engine PubMed with the subjects “Fontan circulation”, “cardiac autonomic function”, as well as “Fontan circulation” and “heart rate variability”, see **Supplement 1** for the complete queries.

#### 2.1.2 Exclusion & inclusion criteria

Studies describing cardiac autonomic function and/or heart rate variability in Fontan patients were included. Studies that did not address Fontan patients, cardiac autonomic function, or heart rate variability were excluded.

Two reviewers (E.P. and M.N.) independently screened the literature search results on eligibility for inclusion. Only studies involving patients with a clinically or pathologically confirmed diagnosis were considered. Articles were required to be published in peer-reviewed journals and written in English to be included; studies without an available English text were excluded. A database of the primary search results was created, and a list of non-duplicate studies was compiled based on predefined inclusion and exclusion criteria. Relevant studies cited in the reference lists of the primary search results were also identified and incorporated into the review process. The bibliographies of all included articles and relevant published reviews were examined to ensure that no pertinent studies were omitted during the initial search.

#### 2.1.3 Study endpoints

The primary endpoints extracted from eligible studies were parameters commonly used to assess cardiac autonomic nervous function. These included, yet were not limited to, baroreflex sensitivity (BRS), which reflects vagal control of heart rate in response to blood pressure fluctuations; the low-frequency (LF) to high-frequency (HF) power ratio (LF/HF ratio), a frequency-domain measure derived from HRV used to estimate the balance between sympathetic and parasympathetic tone; and the standard deviation of all normal-to-normal (NN) intervals (SDNN), a time-domain index representing overall HRV and the combined influence of both branches of the autonomic nervous system. An overview of 24-h ambulatory ECG recording-based measurements, including HRV and QT variability, is provided in Supplementary Table 1. These parameters were used as surrogates of autonomic cardiovascular regulation in the Fontan population. To investigate the factors influencing CANA, relevant contextual variables were extracted, including underlying ventricular morphology, surgical stage, time since Fontan completion and type of Fontan procedure. Secondary endpoints included parameters of clinical outcome such as arrhythmias, exercise intolerance, and patient demise.

## 3 Results

**Fig. 2:**
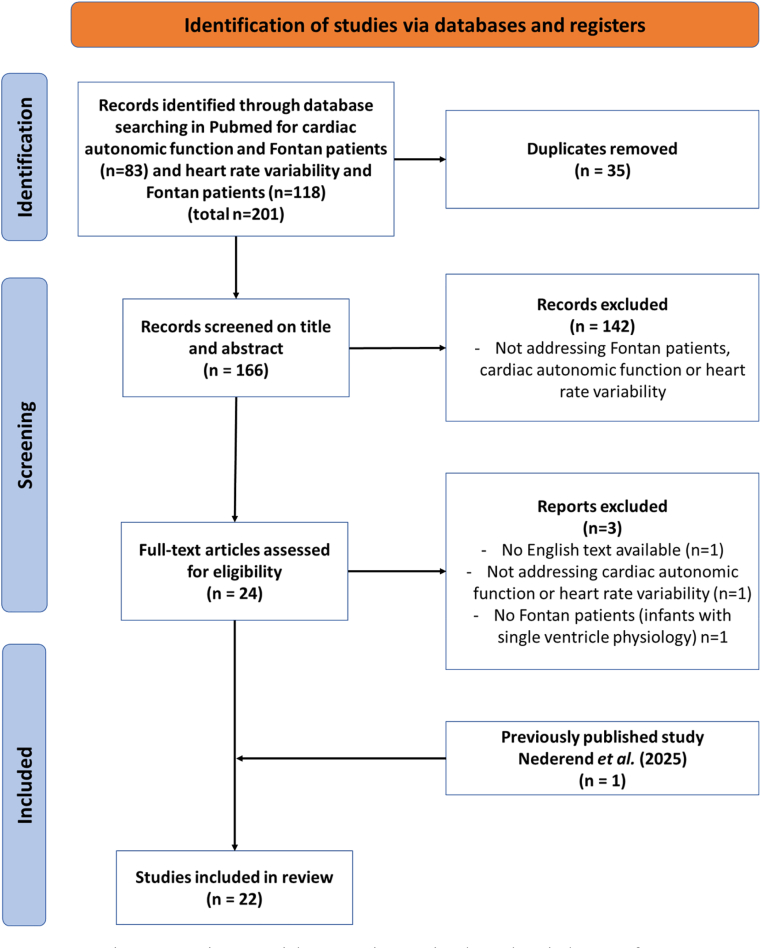
Prisma flow diagram illustrating the selection of studies for literature review. Fontan patients: patients with an univentricular physiology after Fontan palliation.

A total of 201 articles were identified using the search strategy after duplicates were removed. After further screening, 24 articles were selected for full-text evaluation. Of these, 3 were excluded, whereafter 21 articles were selected. In addition, we included one paper on the subject that we recently published [26]. As a result, a total of 22 articles were reviewed for cardiac autonomic function (Fig. 2) [[13], [14], [15],^23^,^24^,[27], [28], [29], [30], [31], [32], [33], [34], [35], [36], [37], [38], [39], [40], [41], [42]]. Epidemiological, anatomical, and clinical details of the included studies are shown in Table 1.

**Table 1:** Epidemiological, anatomical, and clinical details of all included studies on cardiac autonomic function in patients with (functional) univentricular heart disease after Fontan palliation.

	Author and year	Number of Fontan patients	Cohort	Age, years	Age at Fontan operation	Systemic left ventricle	Type of Fontan	Echocardiographic function or functional status
1.	*Bossers* et al.*, 2015* [27]	115	115 Fontan patients, and 32 controls	12 ± 3	3 ± 1	72 (63 %)	EC: 69 (60 %), LT: 46 (40 %)	Mean EF: 52 %
2.	*Butera* et al.*, 1999* [28]	27	39 patients with univentricular physiology (12 with partial CPC) 16 CHD patients with biventricular physiology, and 18 controls	13	6	14 (52 %)	TCPC: 13 (48 %), APC: 14 (52 %)	96 % of patients in NYHA I-II, 4 % in NYHA III
3.	*Dahlqvist* et al.*, 2012* [30]	112	112 Fontan patients, 66 controls	9 ± 4	3 ± 2	49 (44 %)	EC: 44 (39 %), LT: 68 (61 %)	na
4.	*Dahlqvist* et al.*, 2014* [29]	27	27 Fontan patients, and 41 controls	10 [3–17]	2 ± 1	13 (48 %)	TCPC: 27 (100 %)	SVFx: 67 % good, 33 % mildly reduced
5.	*Dahlqvist* et al.*, 2019* [32]	113	113 Fontan patients (12 with pacemaker), and 66 controls	na: paediatric	3 [1–7]	55 (49 %)	EC: 58 (51 %), LT: 55 (49 %)	SVFx: 71 % good, 29 % mildly reduced
6.	*Dahlqvist* et al.*, 2021* [31]	71	89 patients with univentricular physiology, and 38 controls	4 [2–9]	3 [2–10]	28 (32 %)	EC: 68 (96 %), LT: 3 (4 %)	SVFx: 86 % good, 14 % mildly reduced
7.	*Davos* et al.*, 2003* [14]	22	22 Fontan patients, and 22 controls	26 ± 10	13 ± 8	5 (23 %)	TCPC: 3 (36 %), APC: 10 (46 %), AVA: 4 (18 %)	SVFx: 64 % good, 14 % mildly reduced, 9 % moderately to severely reduced
8.	*Eser* et al.*, 2016* [33]	8	8 Fontan patients, and 12 controls	18 ± 5∗	na	6 (75 %)	na	All patients in NYHA I-II
9.	*Fritz* et al.*, 2020* [34]	29	222 CHD patients and 57 controls	29 ± 7	na	na	na	na
10.	*Harteveld* et al.*, 2022* [35]	35	35 Fontan patients, and 34 controls	14 [13–17]	3 [3–4]	21 (60 %)	na	Mean GLS 15.2 %, all patients mildly reduced to good SVFx
11.	*Lambert* et al.*, 2013* [36]	18	18 Fontan patients, and 23 controls	25 ± 1	4 [3–7]	13 (72 %)	EC: 1 (6 %), LT: 8 (44 %), APC: 9 (50 %)	SVFx: 67 % good, 33 % mildly reduced
12.	*Madan* et al.*, 2014* [37]	19	46 patients with univentricular physiology	na: paediatric	9 ± 6	12 (63 %)	EC: 8 (42 %), LT: 11 (58 %)	na
13.	*Nederend* et al.*, 2025* [26]	54	54 Fontan patients	22 [20–27]	3.5 [3–5]	27 (50 %)	EC: 43 (80 %), LT: 5(9 %), APC: 6(11 %)	Mean EF: 47 ± 7 %
14.	*Ohuchi* et al.*, 2001* [15]	63	63 Fontan patients, and 44 controls	13	7	19 (31 %)	TCPC: 45 (71 %), APC: 18 (29 %)	Mean EF: 50 %
15.	*Ohuchi* et al.*, 2004* [24]	97	97 Fontan patients, and 48 controls	14	7	44 (45 %)	EC: 16 (16 %), LT: 59 (61 %), APC: 22 (23 %)	30 % of patients in NYHA I, 60 % in II, and 10 % in III-IV
16.	*Ohuchi* et al.*, 2005* [38]	27	124 (postoperative) CHD patients, and 11 controls	14 ± 6	na	na	TCPC: 17 (63 %), APC: 10 (37 %)	Mean EF: 51 %
17.	*Ohuchi* et al.*, 2007* [39]	42	42 Fontan patients, and 12 controls	15 ± 4	na	17 (40 %)	TCPC: 26 (62 %), APC 16 (38 %)	Mean EF: 52 %
18.	*Ohuchi* et al.*, 2011* [23]	91	292 (postoperative) CHD patients	14 ± 6	6 ± 4	37 (41 %)	TCPC: 77 (85 %), APC: 14 (15 %)	30 % of patients in NYHA I, 59 % in II, and 11 % in III-IV
19.	*Okólska* et al.*, 2021* [40]	30	30 Fontan patients, and 30 controls	24 ± 5	na	24 (80 %)	TCPC: 29 (96 %), APC: 1 (4 %)	17 % of patients in NYHA I, 71 % in II, and 12 % in III
20.	*Rydberg* et al.*, 2004* [13]	10	10 Fontan patients, and 24 controls	10 [3–18]	7 [2–13]	8 (80 %)	TCPC: 10 (100 %)	SVFx: 60 % good, 40 % mildly reduced
21.	*Rydberg* et al.*, 2005* [42]	13	13 Fontan patients, and 37 controls	5†	6 [2–14]	10 (77 %)	TCPC: 13 (100 %)	SVFx: 85 % good, 15 % mildly reduced †
22.	*Rydberg* et al.*, 2008* [41]	15	15 Fontan patients	na: paediatric	5 [1–12]	8 (53 %)	TCPC: 15 (100 %)	SVFx: 86 % good, 7 % mildly reduced, 7 % moderately reduced

∗ time since Fontan completion, † at first follow-up (out of three consecutive follow-up moments).

AP: atriopulmonary connection, AVA: atrioventricular anastomosis, CANA: cardiac autonomic nervous activity, CHD: congenital heart disease, CPC: cavopulmonary connection, EC: extra cardiac conduit, EF: ejection fraction, GLS: global longitudinal strain, HR: heart rate, HRV: heart rate variability, LT: lateral tunnel, na: not applicable or specified, NYHA: New-York Heart association functional class, SND: sinus node dysfunction, SVFx: estimated systemic ventricle function, TCPC: total cavopulmonary connection.

### 3.1 CANA in Fontan patients

Most of the selected studies focused on pediatric Fontan patients [13,15,23,24,[27], [28], [29],^31^,^32^,^35^,[37], [38], [39],[41], [42], [43]], with only six reporting on adult Fontan patients [14,26,33,34,36,40]. The majority of these included asymptomatic Fontan patients with relatively preserved systemic ventricular function.

**Fig. 3:**
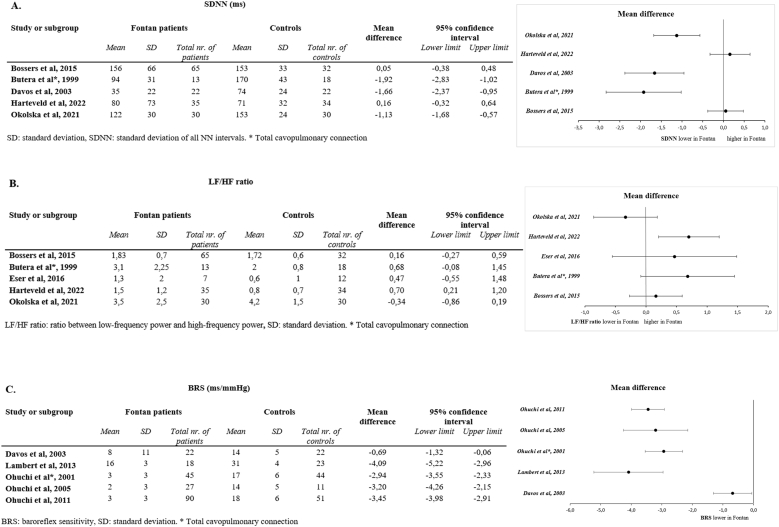
Forest plot for (A) standard deviation of all NN intervals, (B) ratio between low-frequency power and high-frequency power and (C) baroreflex sensitivity in Fontan patients vs healthy controls. BRS: baroreflex sensitivity, LF/HF ratio: ratio between low-frequency power and high-frequency power**,** SD: standard deviation, SDNN: standard deviation of all NN intervals.

Reported alterations in cardiac autonomic nervous activity were compiled and analysed to characterize general patterns observed in the Fontan population. The parameters of CANA and the main findings per study are shown in Table 2, Table 3. Most studies used HRV parameters, based on the frequency domain in 24-h Holter recordings or shorter ECG-based measurements [[13], [14], [15],^23^,^24^,[26], [27], [28], [29], [30], [31], [32], [33], [34],[37], [38], [39], [40], [41], [42]]. In addition, several studies performed complementary modalities, including baroreflex sensitivity testing [14,15,23,24,36,38,39], head-up tilt to examine orthostatic adaptation [35,36], direct muscle sympathetic nerve activity recording [36] and exercise testing with HRR or chronotropic index [26,33,34]. Decrease in autonomic function within the Fontan population as compared to healthy controls was consistently reported in studies which employed standardized, longer-duration measurement protocols. In particular, noticeable changes were marked in key metrics indicative of autonomic dysregulation, such as the mean and standard deviation of NN intervals, LF/HF power ratio, and BRS. This may suggest an imbalance in sympathovagal modulation, characterized predominantly by decreased parasympathetic tone. A consistent trend towards autonomic impairment is illustrated by a forest plot depicting the aggregated mean differences and standard deviations for these parameters across studies (Fig. 3). In selected papers, SDNN was consistently decreased in Fontan patients compared to healthy controls, with reductions of −1.1 to −1.9 standard units reported in both pediatric [28] and adult cohorts [14,40] (Fig. 3A). The reductions correspond to a 20–30 % loss of HRV, linking it to autonomic dysfunction regardless of the age group. In the remaining two pediatric studies [27,35], no significant difference was detected, which may relate to a smaller sample size or methodological variability. Regarding the LF/HF ratio, in pediatric cohorts [27,28,33,35], the overall values were higher compared to those of healthy controls, consistent with relative sympathetic predominance, although only one study reached statistical significance [35] (Fig. 3B). In contrast, in one study in adults [40], slightly lower values compared to healthy controls were reported. The inconsistency may reflect the limited reliability of the LF/HF ratio as a marker of autonomic dysfunction and possibly the sensitivity to methodological conditions.

**Table 2:** Aim, methods and main findings of all included studies on cardiac autonomic function in patients with (functional) univentricular heart disease after Fontan palliation.

	Author and year	Primary aim	Type of assessment	Main findings in relation to CANA/HRV	Extra findings
1.	*Bossers* et al.*, 2015* [27]	To evaluate HR and rhythm abnormalities in Fontan patients and compare the LT and EC technique.	24-h holter recordings: time and frequency domain	In Fontan patients (in comparison to controls) the LF, HF and pNN50 is significantly lower. The LF/HF ratio is significantly higher in EC in comparison to LT technique.	Arrhythmia incidence is low in Fontan patients, yet higher in LT technique. Incidence of SND is high, with no difference in surgical technique.
2.	*Butera* et al.*, 1999* [28]	To determine whether CPC are associated with abnormalities in HRV.	24-h holter recordings: time and frequency domain	Patients with CPC have significantly reduced HRV and a particularly low vagal drive in comparison to healthy controls and biventricular repair patients.	Biventricular repair showed no difference in HRV in comparison to controls. Partial CPC leads to less altered HRV, potentially EC does also.
3.	*Dahlqvist* et al.*, 2012* [30]	To investigate HRV, focusing on the relation between HRV and surgical procedure in Fontan patients.	24-h holter recordings: time and frequency domain	In Fontan patients the HRV was significantly reduced in comparison to controls, no difference between EC or LT technique.	The reduction in HRV was progressive over time.
4.	*Dahlqvist* et al.*, 2014* [29]	To evaluate the intermittent use of a handheld ECG system for detecting silent arrhythmias and cardiac autonomic dysfunction in Fontan patients.	Hand-held ECG devices during 30 s and Poincaré plots	Fontan patients did not show any statistically significant differences in HRV parameters compared to the controls.	The handheld ECG system was good to detect asymptomatic arrhythmia.
5.	*Dahlqvist* et al.*, 2019* [32]	To determine whether changes in HRV could be detected by 24-h holter recordings in Fontan patients with SND.	24-h holter recordings: frequency domain and Poincaré plots	Fontan patients without SND had lower HRV parameters in comparison to controls. Fontan patients with SND, showed higher HRV than both Fontan patients without SND and healthy controls.	Patients requiring a pacemaker due to SND had pre-implantation 24-h ECG recordings showing a tendency toward decreased HRV compared to Fontan patients and SND without pacemakers.
6.	*Dahlqvist* et al.*, 2021* [31]	To investigate changes in HRV during staged TCPC, and compare with age-matched healthy controls.	24-h holter recordings: frequency domain and Poincaré plots	Patients with TCPC showed reduced HRV compared with healthy controls. This reduction was progressive throughout the consecutive stages of TCPC.	HRV was reduced after BDG procedure, and further reductions of HRV were seen post-TCPC.
7.	*Davos* et al.*, 2003* [14]	To assess several aspects of CANA late after the Fontan procedure and examine its relationship with cardiac physiology and clinical status.	20-min ECG: time and frequency domain	CANA is markedly depressed in patients late after the Fontan operation, with both HRV and BRS reduced by >50 %. Patients with marked right atrial dilation had more suppression of their sympathetic compared with the parasympathetic system.	Stronger baroreflexes seem to be associated with a higher (rather than lower) incidence of sustained atrial tachyarrhythmia.
8.	*Eser* et al.*, 2016* [33]	To quantify differences in CANA at rest and after an orthostatic challenge by means of HRV, and HR recovery after an exercise test between adult Fontan patients and age- and gender matched healthy controls.	4-min ECG: frequency domain, CPET	In both supine and standing position, frequency domain HRV parameters were reduced in Fontan patients compared to controls, while there were no differences in mean RR-interval and LF/HF ratio between the groups.	Relative response in HRV parameters to orthostatic challenge was not reduced in Fontan patients.
9.	*Fritz* et al.*, 2020* [34]	To analyse the parasympathetic activity (by HRV), in patients with CHD according to diagnostic subgroups and severity in comparison to healthy controls, and to evaluate the association between the parasympathetic activity and exercise capacity.	ECG recording of 130 consecutive beats: time domain, CPET	Patients with CHD showed impairments in the parasympathetic activity compared with healthy controls, patients with Fontan circulation were most impaired.	The impaired parasympathetic activity is associated with reduced exercise capacity.
10.	*Harteveld* et al.*, 2022* [35]	To unravel the adaptation to orthostatic stress in paediatric Fontan patients by non-invasively examining the cardiovascular and autonomic response to HUTT.	VU-ams monitor system: time and frequency domain, HUTT	Paediatric Fontan patients can respond adequately to orthostatic stress and maintain adequate BP, cardiac output and cerebral blood flow. There was no evidence of autonomic circulatory impairment.	Enalapril (ACE-i) did not influence the response.
11.	*Lambert* et al.*, 2013* [36]	To investigate direct recording of sympathetic nerve traffic through microneurography, endothelial function and arterial stiffness in Fontan patients and matched healthy controls to determine their relative contribution in the changes of SVR in patients with Fontan circulation.	Direct muscle sympathetic nerve activity recording, sympathetic and cardiac baroreflex function, HUTT	Adult Fontan patients have increased activity of the peroneal nerve (direct measurement, represents sympathetic nerve activation) and altered endothelial function. These factors may contribute to the increased SVR.	The magnitude of this increase (levels 60 % greater than those in healthy control subjects) is similar to that observed in patients with heart failure compared to controls.
12.	*Madan* et al.*, 2014* [37]	To compare the bidirectional superior cavopulmonary anastomosis (with preserved APBF) and the TCPC, with regard to their effects on CANA as measured by HRV indices prior to and early after surgery.	15-min ECG: time and frequency domain	TCPC leads to a significant reduction in overall CANA, compared to bidirectional superior cavopulmonary anastomosis with APBF.	
13.	*Nederend* et al.*, 2025* [26]	To evaluate cardiac autonomic function using heart rate variability and exercise testing, and to investigate its association with atrial arrhythmias in a contemporary cohort of adult Fontan patients	24-h ambulatory ECG (time- and frequency-domain HRV analysis), exercise testing (heart rate reserve, chronotropic index)	Patients with atrial arrhythmias had significantly lower SDNN and SDANN (independent of beta-blocker use), indicating impaired autonomic regulation; HRR and chronotropic index were also reduced	Atrial arrhythmias were present in 26 % of patients; ventricular arrhythmias in 7 %. Lower HRR and chronotropic index partly attributable to beta-blocker use. Impaired autonomic function may precede late complications
14.	*Ohuchi* et al.*, 2001* [15]	To evaluate CANA in Fontan patients and to evaluate if the type of surgical procedure is of influence on CANA.	5-min ECG: frequency domain, baroreflex sensitivity, cardiac parasympathetic nervous tone, postsynaptic β-sensitivity	All CANA indexes, except postsynaptic β-sensitivity, in both Fontan groups were significantly lower than in controls, and plasma norepinephrine level was significantly higher in Fontan groups than in controls. There was no difference in CANA indexes between APC and TCPC groups.	None of the CANA indexes was correlated with the number of surgical procedures, age at the time of Fontan operation, or length of follow-up.
15.	*Ohuchi* et al.*, 2004* [24]	To measure various NHA and CANA indices and compare the results with clinical status, including hemodynamic and cardiopulmonary capacity in Fontan patients.	5-min ECG: frequency domain, baroreflex sensitivity	All CANA indices were markedly abnormal; however, they could not differentiate between functional classification. There was no difference in CANA indices between symptomatic and asymptomatic patients, between low- and high-age groups, or between the APC and the TCPC patients. Although symptomatic Fontan patients exhibit higher NHA, CANA is not related to either NYHA class or NHA. Symptomatic Fontan had higher NHA (ANP, BNP, NE, PRA renal activity).	NYHA class, EF, SaO2, and pVO2 were lower and VE-VCO2 slope was higher in the right ventricular type than the left ventricular type Fontan patients. However, ventricular morphology had no influence on the CANA or NHA indices, except for higher NE in the right ventricular type group. APC itself is responsible for higher natriuretic peptides, and arterial oxygen desaturation has a great impact on elevated NHA in the TCPC patients.
16.	*Ohuchi* et al.*, 2005* [38]	To evaluate NCR in CHD patients and correlate this with CANA.	5-min ECG: frequency domain, baroreflex sensitivity, cardiac parasympathetic nervous tone, postsynaptic β-sensitivity	In Fontan patients, the HRV is significantly reduced in comparison to simple and complex (biventricular) heart disease and controls.	In the Fontan patients, all CANA indices correlated with NCR. However, there was no correlation with NHA or hemodynamic variables (in contrast to biventricular CHD).
17.	*Ohuchi* et al.*, 2007* [39]	To evaluate the relationship between central hypercapnic chemosensitivity, CANA and clinical status in Fontan patients.	5-min ECG: frequency domain, baroreflex sensitivity	No difference in CANA (except higher NE levels in high group) between central hypercapnic chemosensitivity high or normal group.	One quarter of young Fontan patients showed an increased central hypercapnic chemosensitivity and this was associated with sympathetic activation and closely related to the accelerated rest and exercise ventilation, although the central hypercapnic chemosensitivity had no relation to exercise capacity.
18.	*Ohuchi* et al.*, 2011* [23]	To clarify the prognostic value of the CANA variables in postoperative CHD patients after biventricular and Fontan repairs.	5-min ECG: frequency domain, baroreflex sensitivity	In Fontan patients, no CANA variables, except for the plasma NE level, predicted future clinical events.	All CANA variables, especially the BRS, were useful predictors for future clinical events in biventricular CHD patients
19.	*Okólska* et al.*, 2021* [40]	To assess the relationship between HRV parameters, exercise capacity, and multiorgan complications in adult Fontan patients	24-h holter recordings: time and frequency domain	Fontan patients had significantly reduced HRV, with a correlation between HRV parameters and age at the time of surgical intervention, time since operation, reduced exercise capacity (lower VO2 peak and percentage of predicted value of VO2 peak, and higher VE/VCO2), and organ complications (GGT and pNN50).	GGT, U/L: 61.5 (44.0–117.0) in Fontan patients vs 15.5 (14.0–18.0) in controls.
20.	*Rydberg* et al.*, 2004* [13]	To examine HRV in Fontan patients and the change in HRV over time.	24-h holter recordings: time and frequency domain	Fontan patients older than 10 years have reduced HRV. However, in patients younger than 10 years, there were only minor differences compared to controls.	Patients with TCPC show a progressive reduction of HRV parameters over time.
21.	*Rydberg* et al.*, 2005* [42]	To assess longitudinal changes in HRV in Fontan patients.	24-h holter recordings: time and frequency domain	Fontan patients had reduced HRV. A significant difference was found between patients and their controls with respect to HF at the second, and third examination. The LF/HF progressively increased in those with the Fontan circulation.	Holter recordings can detect a progressive sympatovagal imbalance and bradycardia over time. Bradycardia is common in Fontan patients.
22.	*Rydberg* et al.*, 2008* [41]	To investigate HRV in Fontan patients, recorded before onset of arrhythmias, to determine whether frequency domain analysis or Poincaré plots can predict the development of arrhythmias.	24-h holter recordings: time and frequency domain, Poincaré pots	There was no difference between patients with arrhythmia and without arrhythmia in terms of HRV parameters, although there was a trend for LF and HF. The tendency towards higher HRV parameters (TP, VLF, LF, and HF) were stronger at a mean of 4.6 months before the onset of arrhythmias compared to 1 year before the onset of arrhythmias.	There was a statistically significant difference in SD1 in the arrhythmia group and had different Poincaré plots (complex or rhomboid-shaped).

ACE-i: angiotensin-converting enzyme inhibitors, APBF: antegrade pulmonary blood flow, BP: blood pressure, BRS: baroreflex sensitivity, BDG: bidirectional Glenn procedure, CANA: cardiac autonomic activity, CHD: congenital heart disease, CPC: cavopulmonary connection, EC: extracardiac conduit, EF: ejection fraction, GGT: gamma-glutamyl transpeptidase, HF: high frequency power, HRV: heart rate variability, HUTT: head-up tilt testing, LF: low frequency power, LT: lateral tunnel, NCR: negative chronotropic response, NHA: neurohormonal activity, NYHA class: New-York Heart Association functional class, pVO_2_: peak VO_2_, SaO_2_: oxygen saturation, SD1: standard deviation of Poincaré plot perpendicular to the line-of-identity, SND: sinus node dysfunction, SVR: systemic venous resistance, TCPC: total cavopulmonary connection, TP: total power, VE-VCO_2_: ventilation/carbon dioxide production slope.

**Table 3:** Parameters of cardiac autonomic function in patients with (functional) univentricular heart disease after Fontan palliation. Articles without absolute (logarithmic) values given per group (Fontan and/or control) are not shown.

	Time domain					Frequency domain					Baroreflex
	Mean NN, ms	SDNN, ms	SDANN, ms	rMSSD, ms	pNN50, %	VLF, ms^2^	LF, ms^2^	HF, ms^2^	TP, ms^2^	LF/HF ratio	BRS, ms/mmHg
*Bossers* et al.*, 2015* [27]											
Fontan	765 ± 118	156 [118–207]	138 [110–181]	36 [23–65]	11 [4–25]∗	1564 [739–2981]	637 [239–1351]∗	344 [117–1031]∗	2532 [1165–5680]	1.83 [1.36–2.29]	–
Controls	722 ± 20	153 [144–188]	129 [120–163]	47 [39–58]	20 [14–30]	1708 [1084–2420]	1303 [702–1706]	717 [361–1254]	3740 [2138–4940]	1.72 [1.33–2.09]	–
*Butera* et al.*, 1999* [28]											
TCPC	698 ± 99	94 ± 31∗	–	22.6 ± 9.7∗	3.6 ± 4.8∗	–	159 ± 210∗	65 ± 117∗	760 ± 980∗	3.1 ± 2.25	–
APC	752 ± 64	121 ± 31∗	–	29 ± 22∗	6 ± 9∗	–	176 ± 156∗	98 ± 120∗	972 ± 718∗	3.0 ± 2.0	–
Controls	845 ± 134	170 ± 43	–	75 ± 36	32 ± 13	–	1562 ± 1418	1443 ± 2039	6550 ± 5000	2.0 ± 0.8	–
*Dahlqvist* et al.*, 2012* [30] ‡											
Fontan	–	–	–	–	–	3.2 ± 0.04∗	2.9 ± 0.08∗	2.7 ± 0.06∗	3.5 ± 0.04∗	0.18 ± 0.02∗	–
Controls	–	–	–	–	–	3.3 ± 0.04	3.1 ± 0.05	3 ± 0.07	3.7 ± 0.05	0.08 ± 0.03	–
*Davos* et al.*, 2003* [14]											
Fontan	–	35 ± 22∗	–	31 ± 30∗	13 ± 22∗	855 ± 2056	254 ± 401∗	263 ± 668∗	–	–	8 ± 11
Controls	–	74 ± 24	–	65 ± 30	36 ± 22	847 ± 497	788 ± 506	853 ± 682	–	–	14 ± 5
*Eser* et al.*, 2016* [33]†											
Fontan	812 ± 91	–	–	–	–	–	104 [301]∗	58 [154]∗	244 [540]∗	1.3 [3.0]	–
Controls	934 ± 185	–	–	–	–	–	641 [524]	720 [892]	1573 [856]	0.6 [1.6]	–
*Fritz* et al.*, 2020* [34]											
	–	–	–	27 ± 26∗	–	–	–	–	–	–	–
	–	–	–	65 ± 35	–	–	–	–	–	–	–
*Harteveld* et al.*, 2022* [35]†											
Fontan	–	80 [28–126]	–	61 [21–107]	–	–	1296 [67–4555]	944 [32–2467]	–	1.5 [0.8–2.4]	–
Controls	–	71 [55–98]	–	30 [23–37]	–	–	1229 [570–1788]	1235 [509–3045]	–	0.8 [0.4–1.4]	–
*Lambert* et al.*, 2013* [36]											
	–	–	–	–	–	–	–	–	–	–	16 ± 3∗
	–	–	–	–	–	–	–	–	–	–	31 ± 4
*Madan* et al.*, 2014* [37]											
Fontan	–	23 [19–33]	–	14 [11–19]	0.1 [0–1.2]	88 [55–171]	35 [19–142]	34 [16–91]	156 [108–378]	1.3 [0.7–1.7]	–
*Nederend* et al.*, 2025* [26]											
Fontan	–	171 ± 78∗	147 ± 65∗	53 [30–102]	16 [4–35]	778 [293–2368]	219 [73–754]	47 [11–317]	1425 [562–5097]	3.4 [2.3–7.6]	–
*Ohuchi* et al.*, 2001* [15]*‡*											
APC	–	–	–	–	–	–	1.4 ± 0.5∗	1.1 ± 0.7∗	–	–	3 ± 3∗
TCPC	–	–	–	–	–	–	1.7 ± 0.5∗	1.4 ± 0.5∗	–	–	3 ± 3∗
Controls	–	–	–	–	–	–	2.5 ± 0.4	2.5 ± 0.5	–	–	17 ± 6
*Ohuchi* et al.*, 2005* [38]*‡*											
Fontan	–	–	–	–	–	–	1.5 ± 0.5∗	1.2 ± 0.5∗	–	–	2 ± 3∗
Controls	–	–	–	–	–	–	2.4 ± 0.2	2.5 ± 0.3	–	–	14 ± 5
*Ohuchi* et al.*, 2011* [23]*‡*											
Fontan	–	–	–	–	–	–	1.6 ± 0.5∗	1.3 ± 0.6∗	–	–	3 ± 3∗
Controls	–	–	–	–	–	–	2.5 ± 0.4	2.5 ± 0.5	–	–	18 ± 6
*Okolska* et al.*, 2021* [40]											
Fontan	922 ± 158∗	122 ± 30∗	112 ± 32∗	17 [11–34]∗	7 [3–13]∗	302 [13–491]∗	333 [93–551]∗	140 [46–303]∗	861 [1–1738]∗	3.5 ± 2.5∗	–
Controls	772 ± 59	153 ± 24	134 ± 25	33 [27–44]	12 [7–13]	492 [256–712]	712 [538–1129]	289 [157–370]	1619 [957–2031]	4.2 ± 1.5	–
*Rydberg* et al.*, 2004* [13]											
Fontan	703	149	–	–	11	1706	916	926	3548	–	–
Controls	642	138	–	–	15	1862	1322	1101	4285	–	–

All data is shown as median [Q1-Q3], median [IQR], or mean ± SD.

∗p < 0.05 in comparison to healthy controls ‡ Logarithmic transformation † Supine measurements [IQR].

APC: atriopulmonary connection, BRS: baroreflex sensitivity, LF: low frequency power, HF: high frequency power, Q: quartile, rMSSD: square root of the mean squared differences of successive NN intervals, SD: standard deviation, SDNN: standard deviation of all NN intervals, SDANN: standard deviation of the average NN intervals calculated over 5-min intervals, TCPC: total cavopulmonary connection, TP: total power, pNN50: percentage of adjacent NN intervals that differ by > 50 ms, VLF: very low frequency power.

On the other hand, BRS demonstrated the most consistent and significant impairment across both pediatric [15,23,38] and adult [36] Fontan populations, with mean differences of −3 to −4 ms/mmHg, corresponding to more than 50 % reduction in reflexive vagal control (Fig. 3C).

Taken together, these findings suggest that parasympathetic withdrawal and impaired baroreflex function are core factors of the Fontan circulation across age groups.

### 3.2 Relation of CANA with clinical outcomes

Several studies reported on a possible relationship of CANA with clinical outcomes [14,23,24,34,38,40]. Ohuchi et al., in separate studies involving 97 and 27 pediatric Fontan patients, respectively, found that although all CANA parameters were markedly abnormal as compared to controls, they could not differentiate between New York Heart Association (NYHA) functional classification and reported symptoms [24,38]. Specifically, no significant association was found between HRV indices and hemodynamic variables, serum neurohormonal markers, or NYHA class, indicating that autonomic dysfunction might not be a direct indicator of clinical condition [24]. In their later study, they confirmed that, in contrast to CHD patients with a biventricular circulation, no CANA parameter could predict upcoming clinical events in Fontan patients. Instead, only plasma levels of norepinephrine and brain natriuretic peptide (BNP) were associated with an increased risk of adverse clinical outcomes in Fontan patients [23].

A significant decrease in HRV indices such as Root mean square of the successive differences (RMSSD, Table S1) and HF power was observed in adult Fontan patients, based on cohorts of 30 patients [40] and 29 Fontan patients within a larger cohort of 222 CHD patients [34], suggesting progressive autonomic dysfunction in this population. Reduced HRV indices like RMSSD and HF power, elevated LF/HF ratios alongside decreased BRS, and direct measures of sympathetic activity have all consistently shown a persistently increased sympathetic dominance and reduced parasympathetic tone in studies involving adult Fontan patients [14,33,34,36,40]. Okólska et al. reported a markedly impaired HRV in 30 adult Fontan patients compared to 30 healthy controls [40]. In their analysis, lower HRV indices were associated with older age at surgery, diminished exercise capacity and elevated biomarkers such as gamma-glutamyl transferase (GGT) and N-terminal pro-B-type natriuretic peptide (NT-proBNP), which are linked to Fontan-associated liver disease. Furthermore, the study of correlations with chronotropic index in the Fontan group highlighted the association between autonomic dysfunction, exercise limitation, and end-organ complications [40]. Similarly, Fritz et al. reported the association of reduced parasympathetic activity in the Fontan subgroup with diminished exercise performance as well as elevated markers of organ dysfunction, including gamma glutamyl transpeptidase (GGT) and Tumor necrosis factor (TNF-α), implicated in systemic inflammation [34]. In a separate study of 8 adult Fontan patients, heart rate recovery (HRR) after exercise test remained preserved, whereas autonomic dysfunction at rest was significantly impaired, suggesting that vagal reactivation may be maintained under dynamic conditions [33]. Finally, Lambert et al., in their cohort of 18 Fontan adult patients, demonstrated elevated myocardial sympathetic nerve activity (MSNA) compared with healthy controls (24.8 ± 2.4 vs. 16.8 ± 1.8 bursts per minute), accompanied by reduced BRS (16.0 ± 3.3 vs. 30.9 ± 3.7 ms mmHg^−1^) and increased systemic vascular resistance [36], which was considered part of an adaptive mechanism to the Fontan circulation. Although clinical outcomes were not directly assessed, the authors noted that sympathetic dominance and baroreflex impairment may contribute to Fontan failure [36].

Regarding arrhythmias, Davos et al. reported that 14 out of 22 adult Fontan patients had sustained atrial tachyarrhythmias, which were paradoxically associated with higher baroreflex gain [14]. Overall, BRS was reduced by more than 50 % in most patients, and LF power was inversely correlated with right atrial area, which may imply structural contributions to altered autonomic regulation. Furthermore, LF and total power were significantly lower in patients with higher central venous pressure and worse exercise capacity, suggesting a link between autonomic dysfunction, hemodynamics, and functional status [14]. In addition, we recently reported on differences in autonomic function in adult Fontan patients with versus without atrial arrhythmias, and found that SDNN and standard deviation of the average NN intervals calculated from all 5-min intervals (SDANN) were significantly reduced in patients with atrial arrhythmias [26], indicating decreased autonomic adaptability and regulation in these patients.

#### 3.2.1 Impact of the stage of the Fontan operation

While multiple studies found that the impairment in CANA parameters is progressive during stages of the Fontan operation, as well as over time after Fontan palliation [13,28,31,37,42], one longitudinal study has also assessed pre-operative autonomic state [31]. Evidence indicates that autonomic dysfunction is already present before the Glenn stage. In their cohort of 89 pediatric patients, Dahlqvist et al. reported markedly decreased time- and frequency-domain HRV parameters before the bidirectional Glenn procedure compared with healthy controls [31]. It was proposed that the impairment may reflect both intrinsic autonomic dysfunction and the effect of single ventricle volume overload prior to cavopulmonary connection. After bidirectional Glenn, RR intervals and SD2 (standard deviation along the long axis of the Poincaré plot, Table S1) increased, suggesting longer cycle lengths and greater long-term variability, which may be attributed to the development of sinus node dysfunction in this cohort. In general, SD1 reflects short-term, vagally mediated variability, whereas SD2 represents long-term variability influenced by both sympathetic and parasympathetic inputs; the SD1/SD2 ratio is therefore considered an index of sympathovagal balance. A SD1/SD2 ratio greater than 1 indicates a shift toward vagal dominance, whereas a ratio below 1 reflects sympathetic predominance. Following total cavopulmonary connection (TCPC), however, Dahlqvist et al. reported reductions in total power, LF power and HF power, consistent with overall diminished autonomic function. In addition, Fontan patients had decreased SD1 and a reduced SD1/SD2 ratio [31]. Madan et al. in a cohort of 46 pediatric patients (27 undergoing bidirectional Glenn and 19 TCPC), presented a comparative analysis of surgical stages that showed declines in HRV indices after surgery in both groups, with a more pronounced effect in the TCPC group, suggesting that the final stage may exacerbate autonomic imbalance [37]. Furthermore, serial assessments in a cohort of 13 children after Fontan palliation showed persistently reduced HF power and a progressive increase in the LF/HF, indicating impaired parasympathetic recovery and a shift toward sympathetic dominance [42]. Together, these findings indicate a progressive marked reduction in parasympathetic activity and increased sympathetic influence in pre- and post-Fontan state.

#### 3.2.2 Impact of the type of Fontan operation

Six studies reported on the potential differences in CANA parameters stratified for the surgical techniques used [15,24,27,28,30,37], with some showing significant HRV differences [27,37] and others finding no such variation [15,24,28,30]. Bossers et al., in a cohort of 115 pediatric Fontan patients, compared TCPC subtypes: intra-atrial lateral tunnel and extracardiac conduit technique [27]. The overall incidence of sinus node dysfunction (SND) was 29 %, reporting no significant difference between surgical types. However, a significantly high LF/HF ratio and sinus pauses ≥2 s were exclusively observed in the intra-atrial lateral tunnel group. These patients also demonstrated significantly lower heart rate reserve and impaired HRR post-exercise (p < 0.05), which suggests reduced autonomic responsiveness [27].

In contrast, in the study of Dahlqvist et al. with a cohort of 112 pediatric Fontan patients, LF power, HF power and total power components were significantly lower than in healthy controls (p < 0.05 for all), however, there were no statistically significant differences reported between the lateral tunnel and extracardiac conduit groups [30]. According to Ohuchi et al., all Fontan subtypes showed a significant impairment in HRV, with LF power, HF power (expressed as log-transformed values) and BRS significantly lower than in age-matched healthy controls, independent of operation type [15]. In a subsequent study, Ohuchi et al. confirmed these findings, reporting that surgical type (atriopulmonary connection (APC) vs. TCPC) did not significantly affect autonomic indices [24]. However, ventricular morphology did differ between groups: ANP and BNP levels were significantly higher in the APC group, and right ventricular morphology was more prevalent in these patients. Interestingly, atrial natriuretic peptide (ANP) and brain natriuretic peptide (BNP) levels were higher in patients with APC than in patients with complete TCPC; nevertheless, HRV and BRS were similarly lower in both groups [24]. Butera et al. also observed reduced HRV in patients with both total cavopulmonary and atriopulmonary connections [28]. Additionally, HRV parameters did not depend on clinical status, suggesting that autonomic dysfunction is a feature of the Fontan circulation, independent of symptomatic presentation [28]. Overall, while atrial disruption through APC or lateral tunnel techniques has been hypothesized to be a driver of autonomic dysfunction, autonomic dysfunction has already been reported to be present pre-operatively. After surgery, most of the selected studies detected HRV and BRS impairment across all Fontan subtypes with no consistent difference between surgical types. Moreover, progression of autonomic dysfunction during stages of the Fontan operation and over time after Fontan palliation has been reported. Collectively, these findings support a deterioration in CANA both during Fontan staging and over time, although evidence for variation in CANA parameters between surgical techniques remains scarce.

### 3.3 Gaps in literature

In general, reported studies included relatively small cohorts of patients, indicative of the rare nature of functional single ventricle congenital heart disease. Studies comprising larger international cohorts of >150 patients were not found using our query. We have found several items to be under-addressed in the current literature. Although pediatric cohorts have been assessed at various postoperative stages, only a few studies [15,32] followed patients to adulthood to evaluate how autonomic parameters evolve and how that relates to outcome. Longitudinal data remain scarce, particularly in adults, which makes it difficult to assess the long-term progression as well as the prognostic clinical implications of autonomic impairment beyond childhood and adolescence. In addition, we found limited data on the influence of factors like sex, anatomical substrate and lifestyle. Anatomical factors, especially ventricular morphology, received minimal attention concerning autonomic outcomes, with most studies focusing on surgical technique (e.g., lateral tunnel vs. extracardiac conduit) without accounting for the potential impact of underlying cardiac structure. Ohuchi et al. studied a possible relationship with ventricular morphology, yet found no differences in HRV parameters [24]. Despite classifying patients based on dominant ventricular type (left, right, or biventricular), they found no significant differences in HRV measures between the groups. This may suggest that ventricular morphology alone is not a key determining factor in autonomic dysfunction in the Fontan patient population, but rather one of the factors, but the finding may also reflect the relative scarcity of substantial data addressing this.

## 4 Discussion

This review highlights several consistent findings across studies investigating CANA in patients with single ventricle physiology palliated by a Fontan circulation. Fontan patients are reported to have significantly reduced HRV, which indicates chronic impairment of parasympathetic modulation. Findings across selected studies suggest a gradual worsening of autonomic regulation during the establishment of a Fontan circulation, further declining after the bidirectional Glenn and TCPC procedures. Importantly, a previous review on postnatal cardiac autonomic function in pediatric congenital heart disease suggested that ANS function seems to be altered both pre- and post-surgery in children with CHD [22]. Preoperatively, univentricular patients may already have ANS alterations related to loading and heart failure [31], but evidence compared to healthy controls is lacking.

Overall, HRV impairment appears consistent across all Fontan types while specific parameters, such as heart rate reserve and sinus node function, were reported to be influenced by surgical technique (e.g., lateral tunnel vs. extracardiac conduit). This may suggest that CANA impairment is prevalent and potentially progressive in Fontan patients, with a possible link to arrhythmia risk, heart failure and other long-term outcomes. Notably, while right ventricular morphology is generally linked to poorer clinical outcomes in the Fontan population, almost none of the included studies categorized autonomic data by ventricular type. This leaves it unclear whether impaired CANA contributes to this anatomical risk profile. Furthermore, evidence addressing the adult population, sex differences, and modifiable lifestyle factors remains limited.

Several mechanisms have been proposed to explain the markedly impaired CANA observed in patients with a Fontan circulation. It is possible that congenital heart malformations are connected to autonomic imbalance through abnormal embryologic development, such as insufficient contribution of neural crest cells [44], which in turn may increase the risk of myocardial dysfunction and heart failure later in life. On the other hand, autonomic dysfunction may represent an early consequence of myocardial impairment and becomes more pronounced with disease progression. Overall, it remains unclear whether the observed autonomic abnormalities occur before or result from myocardial deterioration, or represent both processes.

Although most studies have been performed in a clinically relatively stable group of Fontan patients with preserved systemic ventricular function, several studies reported on a possible relationship with clinical outcomes [14,23,24,34,38,40]. The negative chronotropic response to atropine, reflecting parasympathetic function, is diminished in complex CHD, including Fontan patients, which is associated with reduced exercise capacity [24,34,38]. As a result, the reduction in exercise tolerance in these patients may well be related to impaired autonomic nervous system function [34,38]. The interplay between impaired CANA, reduced exercise capacity, and clinical outcomes was reported to be influenced by factors such as surgical history and neurohormonal imbalances, as well as by the specific nature of CHD [14,34]. Additionally, in a cohort of 102 adult ACHD patients, Pizarro et al. reported an association of ACHD with HRV impairment, which has prognostic implications for mortality and sudden cardiac death [45]. They further demonstrated significant correlation of N-terminal pro–B-type natriuretic peptide (NT-proBNP) levels with both time- and frequency-domain indices. Although they did not specifically analyse Fontan patients, they reported no differences in HRV variables across ACHD lesion groups. Butera et al. and Ohuchi et al. also noted these impairments relative to CHD patients with simple defects and complex, yet biventricular, defects [28,38]. Ohuchi et al. reported that CANA parameters, though markedly abnormal in paediatric Fontan patients, did not correlate with functional classification, symptoms, neurohormonal activity, or hemodynamic variables, nor could they predict future clinical events [23,24,38]. This lack of correlation may be due to the timing of the 24-h ambulatory ECG recording, as it may have occurred too early in the disease course. We might speculate that autonomic dysfunction precedes clinically overt heart failure, and that if CANA tests were to be repeated after several years, the results would be more overt. In the included studies reporting on adult patients, autonomic dysfunction appeared more severe and has been associated with higher arrhythmia risk, diminished exercise capacity, and mortality risk [14,33,34,36,40]. In our recent study, we have also demonstrated a significant association between reduced HRV and atrial arrhythmias in a contemporary adult Fontan cohort [26].

In conclusion, autonomic dysfunction in Fontan patients is a persistent and potentially progressive condition, characterized by reduced HRV and impaired parasympathetic tone and increased sympathetic tone. Although the dysfunction is well established, its clinical implications remain unclear, since associations with clinical outcomes such as arrhythmia or Fontan failure are inconsistently reported. Current literature gaps include the lack of larger cohorts, longitudinal data in adults, as well as limited understanding of the influence of anatomy, sex and lifestyle factors. Further longitudinal studies are needed to clarify prognostic value and progression of autonomic parameters.

## CRediT authorship contribution statement

**Elizaveta Polyakova:** Writing – review & editing, Writing – original draft, Visualization, Project administration, Methodology, Investigation, Data curation, Conceptualization. **Marieke Nederend:** Writing – review & editing, Writing – original draft, Visualization, Methodology, Investigation. **Philippine Kies:** Writing – review & editing. **Anastasia D. Egorova:** Writing – review & editing, Supervision, Conceptualization. **Monique R.M. Jongbloed:** Writing – review & editing, Writing – original draft, Supervision, Funding acquisition, Conceptualization.

## Declaration of competing interest

The authors declare that they have no known competing financial interests or personal relationships that could have appeared to influence the work reported in this paper.
