# Supplementary material for: Cardiac autonomic function in patients with single ventricle physiology after Fontan palliation: A literature review
Source: Int J Cardiol Congenit Heart Dis. 2025 Oct 30;22:100636. doi: 10.1016/j.ijcchd.2025.100636 (PMC12664356; doi:10.1016/j.ijcchd.2025.100636)
Supplement: Multimedia component 1 [file mmc1.docx]

**Supplemental index**

**Supplement 1** Search strategy for PubMed

***Search strategy 1: Cardiac autonomic nervous system and Fontan patients***

("cardiacs"[All Fields] OR "heart"[MeSH Terms] OR "heart"[All Fields] OR "cardiac"[All Fields]) AND ("autonomic nervous system"[MeSH Terms] OR ("autonomic"[All Fields] AND "nervous"[All Fields] AND "system"[All Fields]) OR "autonomic nervous system"[All Fields] OR "autonomic"[All Fields] OR "autonomical"[All Fields] OR "autonomically"[All Fields] OR "autonomics"[All Fields]) AND ("functional"[All Fields] OR "functional s"[All Fields] OR "functionalities"[All Fields] OR "functionality"[All Fields] OR "functionalization"[All Fields] OR "functionalizations"[All Fields] OR "functionalize"[All Fields] OR "functionalized"[All Fields] OR "functionalizes"[All Fields] OR "functionalizing"[All Fields] OR "functionally"[All Fields] OR "functionals"[All Fields] OR "functioned"[All Fields] OR "functioning"[All Fields] OR "functionings"[All Fields] OR "functions"[All Fields] OR "physiology"[MeSH Subheading] OR "physiology"[All Fields] OR "function"[All Fields] OR "physiology"[MeSH Terms])

AND

("fontan circulation"[tw] OR "fontan circulations"[tw] OR "fontan circulatory"[tw] OR "fontan circulat*"[tw] OR "fontan circuit"[tw] OR "fontan circuits"[tw] OR "fontan circuit*"[tw] OR "Fontan Procedure"[Mesh] OR "Fontan Procedure"[tw] OR "Fontan Procedures"[tw] OR "Fontan Operation"[tw] OR "Fontan Operations"[tw] OR "Fontan Operat*"[tw] OR "Fontan palliation"[tw] OR "Fontan palliated"[tw] OR "cavopulmonary anastomosis"[tw] OR "cavopulmonary anastomoses"[tw] OR "cavopulmonary anastom*"[tw] OR "cavo pulmonary anastomosis"[tw] OR "cavo pulmonary anastomoses"[tw] OR "cavo pulmonary anastom*"[tw] OR "Bidirectional Glenn"[tw] OR "Bidirectional Glenn*"[tw] OR "Cavopulmonary Shunt"[tw] OR "Cavopulmonary Shunts"[tw] OR "Fontan Palliation"[tw] OR "fontan"[tw] OR "fontan*"[all fields] OR "Univentricular Heart"[Mesh] OR "Univentricular Heart"[tw] OR "Univentricular Hearts"[tw] OR "Univentricular Heart*"[tw] OR "Single Heart"[tw] OR "Single Hearts"[tw] OR "Single Heart*"[tw] OR "Single Ventricle"[tw] OR "Single Ventricles"[tw] OR "Single Ventricular"[tw] OR "Single Ventric*"[tw] OR "Hypoplastic left heart "[tw] OR "Hypoplastic right heart"[tw] OR "Hypoplastic left ventricle "[tw] OR "Hypoplastic right ventricle"[tw] OR "Hypoplastic left ventricles "[tw] OR "Hypoplastic right ventricles"[tw] OR "Hypoplastic left ventric*"[tw] OR "Hypoplastic right ventric*"[tw])

***Search strategy 2: Heart rate variability and Fontan patients***

("heart"[All Fields] AND "rate"[All Fields]) OR "heart rate"[All Fields] AND "variability"[All Fields] AND

("fontan circulation"[tw] OR "fontan circulations"[tw] OR "fontan circulatory"[tw] OR "fontan circulat*"[tw] OR "fontan circuit"[tw] OR "fontan circuits"[tw] OR "fontan circuit*"[tw] OR "Fontan Procedure"[Mesh] OR "Fontan Procedure"[tw] OR "Fontan Procedures"[tw] OR "Fontan Operation"[tw] OR "Fontan Operations"[tw] OR "Fontan Operat*"[tw] OR "Fontan palliation"[tw] OR "Fontan palliated"[tw] OR "cavopulmonary anastomosis"[tw] OR "cavopulmonary anastomoses"[tw] OR "cavopulmonary anastom*"[tw] OR "cavo pulmonary anastomosis"[tw] OR "cavo pulmonary anastomoses"[tw] OR "cavo pulmonary anastom*"[tw] OR "Bidirectional Glenn"[tw] OR "Bidirectional Glenn*"[tw] OR "Cavopulmonary Shunt"[tw] OR "Cavopulmonary Shunts"[tw] OR "Fontan Palliation"[tw] OR "fontan"[tw] OR "fontan*"[all fields] OR "Univentricular Heart"[Mesh] OR "Univentricular Heart"[tw] OR "Univentricular Hearts"[tw] OR "Univentricular Heart*"[tw] OR "Single Heart"[tw] OR "Single Hearts"[tw] OR "Single Heart*"[tw] OR "Single Ventricle"[tw] OR "Single Ventricles"[tw] OR "Single Ventricular"[tw] OR "Single Ventric*"[tw] OR "Hypoplastic left heart "[tw] OR "Hypoplastic right heart"[tw] OR "Hypoplastic left ventricle "[tw] OR "Hypoplastic right ventricle"[tw] OR "Hypoplastic left ventricles "[tw] OR "Hypoplastic right ventricles"[tw] OR "Hypoplastic left ventric*"[tw] OR "Hypoplastic right ventric*"[tw])

**Supplementary Table 1**

**Table 1.** 24-hour ambulatory ECG recording based measurements, including heart rate variability, and QT-variability

| **Abbreviation** | **Principle and/or calculation** | **Autonomic Nervous System Association** |
| --- | --- | --- |
| **BRS** | baroreflex sensitivity | Reflects vagal control of heart rate in response to blood pressure fluctuations |
| **SDNN (ms)** | standard deviation of all NN intervals | Parasympathetic and Sympathetic Nervous System Activity |
| **SDANN (ms)** | standard deviation of the average NN intervals calculated over 5-minute intervals | Parasympathetic and Sympathetic Nervous System Activity |
| **pNN50 (%)** | percentage of adjacent NN intervals that differ by>50 ms | Parasympathetic Nervous System Activity |
| **rMSSD (ms)** | square root of the mean squared differences of successive NN intervals | Parasympathetic Nervous System Activity |
| **SD1 (ms)** | Standard deviation of instantaneous beat-to-beat variability (short axis of Poincaré plot) | Parasympathetic activity |
| **SD2 (ms)** | Standard deviation of continuous long-term variability (long axis of Poincaré plot) | Mixed sympathetic and parasympathetic activity |
| **Ptot (ms²)** | Total spectral power across all frequency bands | Overall autonomic activity |
| **PLF (ms²)** | Power in the low-frequency band (typically 0.04–0.15 Hz) | Mixed sympathetic and parasympathetic activity |
| **PHF (ms²)** | Power in the high-frequency band (typically 0.15–0.4 Hz) | Parasympathetic (vagal) activity |
| **VLF (ms^2^)** | very low-frequency power(0.003–0.04 Hz) | Sympathetic Nervous System Activity |
| **LF (ms^2^)** | low-frequency power (0.04–0.15 Hz) | Parasympathetic and Sympathetic Nervous System Activity |
| **HF (ms^2^)** | high-frequency power (0.15–0.4 Hz) | Parasympathetic Nervous System Activity |
| **LF/HF ratio** | ratio between low-frequency power and high-frequency power | Sympathetic/ Parasympathetic balance |
| **Total power (ms^2^)** | the total power | Parasympathetic and Sympathetic Nervous System Activity |
| **Mean QTc** | Mean corrected QT interval according to Bazett, calculated over 5-minute intervals |  |
| **SD QT** | The standard deviation of the mean corrected QT interval according to Bazett, calculated over 5-minute intervals |  |

ms: milliseconds, NN: normal-to-normal

Source: This table is adapted form Nederend et al. [26], distributed under the terms of the [Creative Commons CC-BY](http://creativecommons.org/licenses/by/4.0/) license
